# Viral Envelope Protein 53R Gene Highly Specific Silencing and Iridovirus Resistance in Fish Cells by AmiRNA

**DOI:** 10.1371/journal.pone.0010308

**Published:** 2010-04-23

**Authors:** Yu-Sin Kim, Fei Ke, Xiao-Ying Lei, Rong Zhu, Qi-Ya Zhang

**Affiliations:** 1 State Key Laboratory of Freshwater Ecology and Biotechnology, Institute of Hydrobiology, Chinese Academy of Sciences, Wuhan, China; 2 College of Life Science, Kim Il Sung University, Pyongyang, Democratic People's Republic of Korea; University of Florida, United States of America

## Abstract

**Background:**

Envelope protein 53R was identified from frog *Rana grylio* virus (RGV), a member of the family *Iridoviridae*, and it plays an important role in the virus assembly. Although inhibition of iridovirus major capsid protein (MCP) by small hairpin RNAs (shRNAs) has been shown to cause resistance to viral infection *in vitro*, RNA interference (RNAi) to inhibit aquatic animal virus envelope protein gene product has not been reported.

**Methodology:**

We devised artificial microRNAs (amiRNAs) that target a viral envelope protein gene RGV *53R*. By incorporating sequences encoding amiRNAs specific to *53R* of RGV into pre-miRNA155 (pSM155) vectors, which use the backbone of natural miR-155 sequence and could intracellularly express *53R*-targeted pre-amiRNAs. The pre-amiRNAs could be processed by the RNase III-like enzyme Dicer into 21–25 nt amiRNAs (amiR-53Rs) in fish cell lines. The levels of *53R* expression were analyzed through real-time PCR and RGV virions assembly were observed by electronic microscopy in fish cells transfected with or without amiR-53Rs at 72 h of RGV infection.

**Conclusion/Significance:**

The results argue that viral envelope protein RGV *53R* can be silenced and the virions assembly was deficient *by a*miR-53R-1, and further identified the first amiRNA of envelope protein gene from iridovirus that was able to cause resistance to virus infection in fish cells. The data demonstrate that the viral infection is efficiently suppressed (58%) by amiR-53R-1 targeting positon 36–57 of RGV *53R*. Moreover, electron microscopic observations revealed virion assembly defect or reduced virions assembly capacity was closely correlated to expression of amiR-53R-1. Based on real time PCR of the *Mx* gene, we found no evidence of activation of IFN by amiR-53R-1.

## Introduction

Iridoviruses are one of the significant viral pathogens for aquatic animals that have caused great economic losses in the aquaculture industry worldwide [1]. *Rana grylio* virus (RGV), a member of iridoviruses, causes systemic infectious iridoviral disease in cultured pig frog (*Rana grylio*) [2]. Some RGV genes and their functions have been identified, including deoxyuridine triphosphatase (dUTPase), thymidine kinase (TK), 3β-hydroxysteroid dehydrogenase (3β-HSD) and ERV1 (essential for respiration and vegetative growth 1) [3]–[6]. Recently, an envelope protein that may play an important role in virus assembly, RGV 53R, was identified and characterized from RGV. RGV *53R* has homologues in all sequenced iridoviruses. The structural conserved features shared by iridovirus envelope proteins were also found in RGV 53R. The intracellular distribution and dynamic changes of RGV 53R revealed that it co-localized with the ER components at early stage of post transfection[7]. Therefore, RGV *53R* was selected for further characterization to understand the molecular *pathogenesis* and gene silencing by artificial microRNAs (amiRNAs) in RGV infection.

Discovery of the RNA interference (RNAi) pathway has led to exciting new strategies for developing treatment for viral diseases [8]. To date, siRNAs and miRNAs have been used for silencing expression of the major capsid protein (MCP) encoded by tiger frog virus (TFV) and red seabream iridovirus (RSIV), two iridovirus causing severe disease in aquatic animals [9]–[11]. But the envelope protein of iridovirus has not been demonstrated as a target for RNAi as well as MCP. RNA interference is a process of post-transcriptional sequence specific gene silencing in many eukaryotes. The use of RNAi to inhibit virus, including siRNAs and miRNAs, offers a new approach for controlling viral infections [12], [13]. Artificial miRNAs (amiRNAs) expression plasmid vectors such as pre-miRNA 155-designed shRNAs vectors (pSM155) which were designed to target on ORF of endogenous and exogenous genes have been developed and used for RNA interference [14]–[18]. The plasmid vector pSM155 used in this study to construct plasmids that express amiRNAs was based on the backbone of natural miR-155. And the natural miRNA: miRNA duplex sequence was replaced by the artificial one [19]. The appearance of plasmid-based expression systems that is effective and inexpensive for amiRNAs generation present rational way for the design and expression of 53R targeted amiRNA.

In the present study, we devised amiRNAs of structured 64-nucleotides that targets different positions of RGV *53R* as pre-miRNA, which could be processed by the RNase III-like enzyme Dicer into 21–25 nt miRNAs (amiR-53R-1, amiR-53R-2 and amiR-53R-3 target 36–57, 476–498 or 37–58 oligos position, respectively) [20], to investigate whether viral envelope protein gene silencing and iridovirus *resistance* mediated by the amiRNAs in fish cells.

## Results

### Expression of pSM155-amiR-53Rs

**Figure 1:**
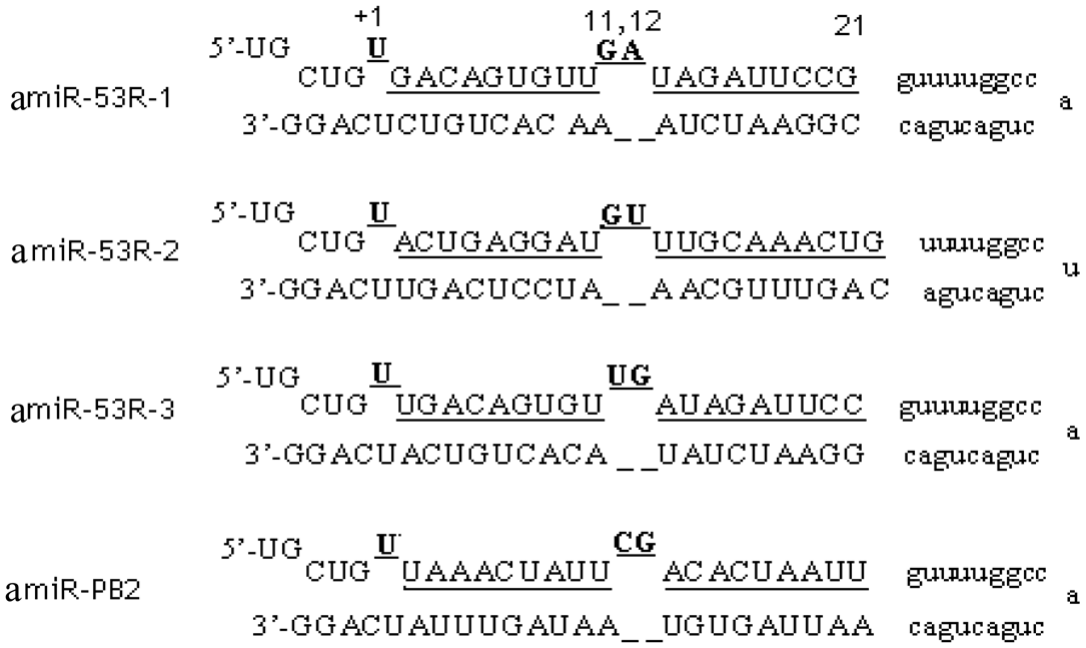
Schematic presentation of predicted stem-loop sequences of pSM155-amiR-53Rs. +1, 11, 12 and 21 are corresponded to positions in stem structure. The antisense of the target sequences for the ORF of *53R* and the 3′ UTR of *PB2* are underlined.

Three pairs of oligonucleotides encoding 53R-specific amiRNAs of RGV (referred as amiR-53R-1, amiR-53R-2 and amiR-53R-3) (Table 1), and a pairs of oligonucleotides corresponding to PB2 gene of avian influenza virus, AIV (referred as amiR-PB2), were annealed and ligated into the pre-miRNA155 (pSM155) vector to create plasmids (pSM155-amiR-53Rs and pSM155-amiR-PB2) capable of producing 53R or PB2 gene encoded pre-amiRNAs in plasmid-transfected cells. The predicted structures of the engineered pre-amiRNAs incorporated into the pSM155 backbone are shown in Fig. 1. When pSM155-amiR-53Rs were transfected into grass carp ovary (GCO) cells, they allowed co-cistronic expression of pre-amiRNAs with GFP in cells under the control of the Pol II human CMV promoter. The co-cistronic expression of the pre-amiRNAs was monitored microscopically under a fluorescence microscope.

**Table 1:** Oligonucleotides sequence encoding 53R-specific pre-miRNAs.

Name	Strand	Oligo sequence	Position of target in gene
amiR-53R-1	Top	TGCTGTGACAGTGTTGATAGATTCCGGTTTTGGCCACTGACTGACCGGAATCTAAACACTGTCT	36–57
	Bottom	CCTGAGACAGTGTTTAGATTCCGGTCAGTCAGTGGCCAAAAC**CGGAATCTATCAACACTGTCA**C	
amiR-53R-2	Top	TGCTGTACTGAGGATGTTTGCAAACTGTTTTGGCCTCTGACTGACAGTTTCAAAATCCTCAGTT	476–498
	Bottom	CCTGAACTGAGGATTTTGAAACTGTCAGTCAGAGGCCAAAA**CAGTTTGCAAACATCCTCAGTT**C	
amiR-53R-3	Top	TGCTGTTGACAGTGTTGATAGATTCCGTTTTGGCCACTGACTGACGGAATCTATACACTGTCAT	37–58
	Bottom	CCTGATGACAGTGTATAGATTCCGTCAGTCAGTGGCCAAAAC**GGAATCTATCAACACTGTCAA**C	

Bold letters represent sense sequences of engineered amiRNAs derived from the target gene.

### Inhibitory effect of amiR-53Rs on 53R expression

**Figure 2:**
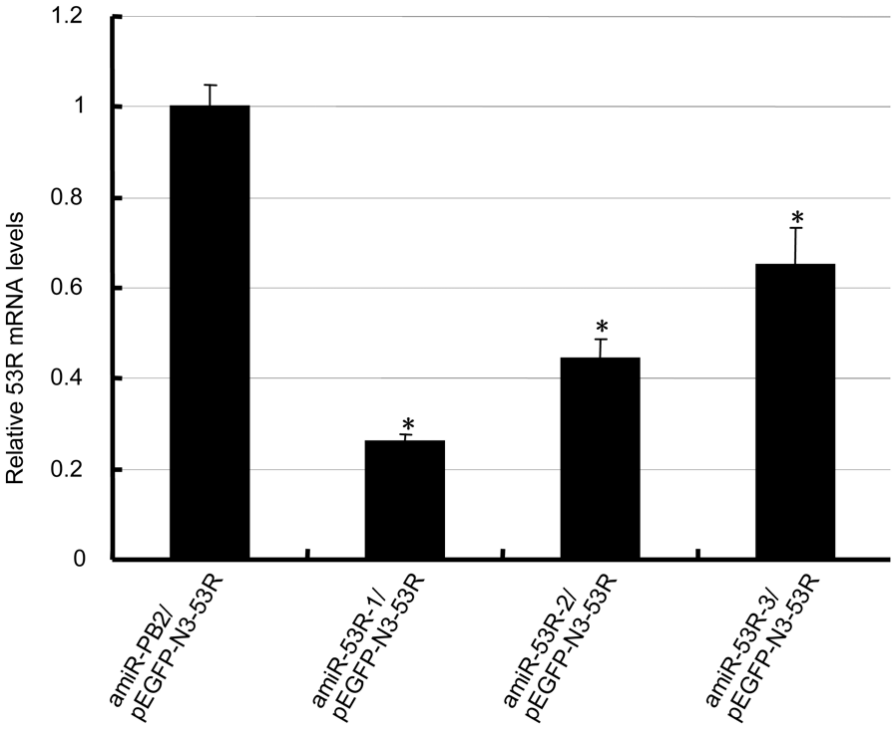
Quantitative analysis of *53R* mRNA levels in cells co-transfected with different plasmids. GCO cells co-transfected with pSM155-amiR-53R-1/pEGFP-N3-53R, pSM155-amiR-53R-2/pEGFP-N3-53R and pSM155-amiR- 53R-3/pEGFP-N3-53R, respectively. *53R* mRNA level from each group was measured by real-time PCR analysis 72 h post transfection. Group co-transfected with pSM155-amiR-PB2/pEGFP-N3-53R was used as negative control. The value of negative control was designated as 1.0 (n = 3). The values represent averages of three independent experiments, with the range indicated (±S.D). *P<0.05 versus control.

When GCO cells were co-transfected with pSM155-amiR-53R-1/pEGFP-N3-53R, pSM155-amiR-53R-2/pEGFP-N3-53R or pSM155-amiR-53R-3/pEGFP-N3-53R, pSM155-amiR- 53Rs silenced the expression of the *53R* gene in different levels compared with the control that were co-transfected with pSM155-amiR-PB2/pEGFP-N3-53R. 53R mRNA levels were evaluated in different groups using the real-time quantitative RT-PCR assay. The results indicated that expression of the *53R* were reduced by 74% (FC = 0.26±0.02), 56% (FC = 0.44±0.04,) and 35% (FC = 0.65±0.08) in cells transfected with pSM155-amiR-53R-1, pSM155-amiR-53R-2 and pSM155-amiR-53R-3 at 72 h, respectively (Fig. 2). The pSM155-amiR-53R-1 was more efficient in inhibiting *53R* gene expression than pSM155-amiR-53R-2 and pSM155-amiR-53R-3. Thus, pSM155-amiR-53R-1 was chosen in our further studies on amiRNAs-mediated inhibition of RGV infection.

### Delayed emergence of CPE of RGV in host cells by amiR-53R-1

Cytopathic effect (CPE) was the most intuitive parameter that reflected the viral quantity of virus accumulation [21]. In GCO cells that were infected with RGV after transfected with pSM155-amiR-53R-1, CPE were markedly delayed than that were transfected with pSM155-amiR-PB2, as well as only infected with RGV.

### Reduction of RGV titer and virions by amiR-53R-1

**Figure 3:**
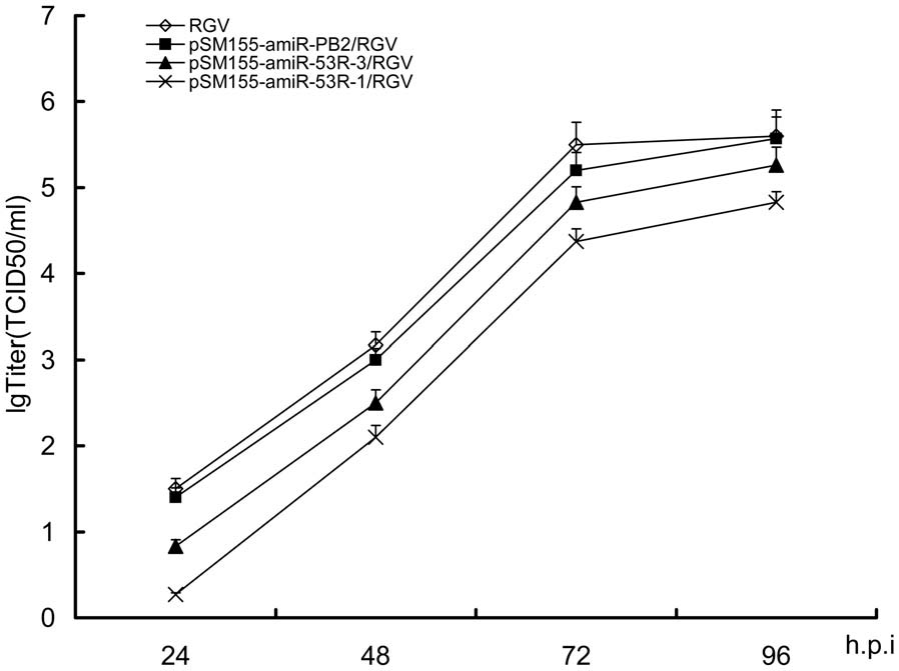
Comparison of virus titers. GCO cells that transfected with pSM155-amiR-53R-1, pSM155-amiR-53R-3 and pSM155-amiR-PB2, respectively, were infected with RGV at 24 h post transfection (MOI of 3). Cell supernatants were collected at 24, 48, 72 and 96 h.p.i. and assayed for virus titration. The titers of RGV in pSM-amiR-53R-1 transfected samples were about 17 -fold, 12-fold, 14-fold and 6-fold lower than those of the transfected with pSM155-amiR-PB2 at different time intervals, respectively. The data show the average titers of three independent experiments in lgTCID_50_ plus S.D.

To test whether pSM155-amiR-53R-1 could impede the packaging and production of infectious RGV, pSM155-amiR-53R-1 transfected cells were infected with RGV (MOI of 3) at 24 h of transfection. Cell supernatants were collected at 24, 48, 72 and 96 h post infection (p.i.) to determine the production of virions. In TCID_50_ assays, the average titers of RGV in pSM155-amiR-53R-1 transfected samples were about 17-fold, 12-fold, 14-fold and 6-fold lower than those of the transfected with pSM155-amiR-PB2 at different time intervals, respectively (Fig. 3). At the same time, the average titers in pSM155-amiR-53R-3 transfected cells were higher than those in pSM155-amiR-53R-1 transfected cells.

**Figure 4:**
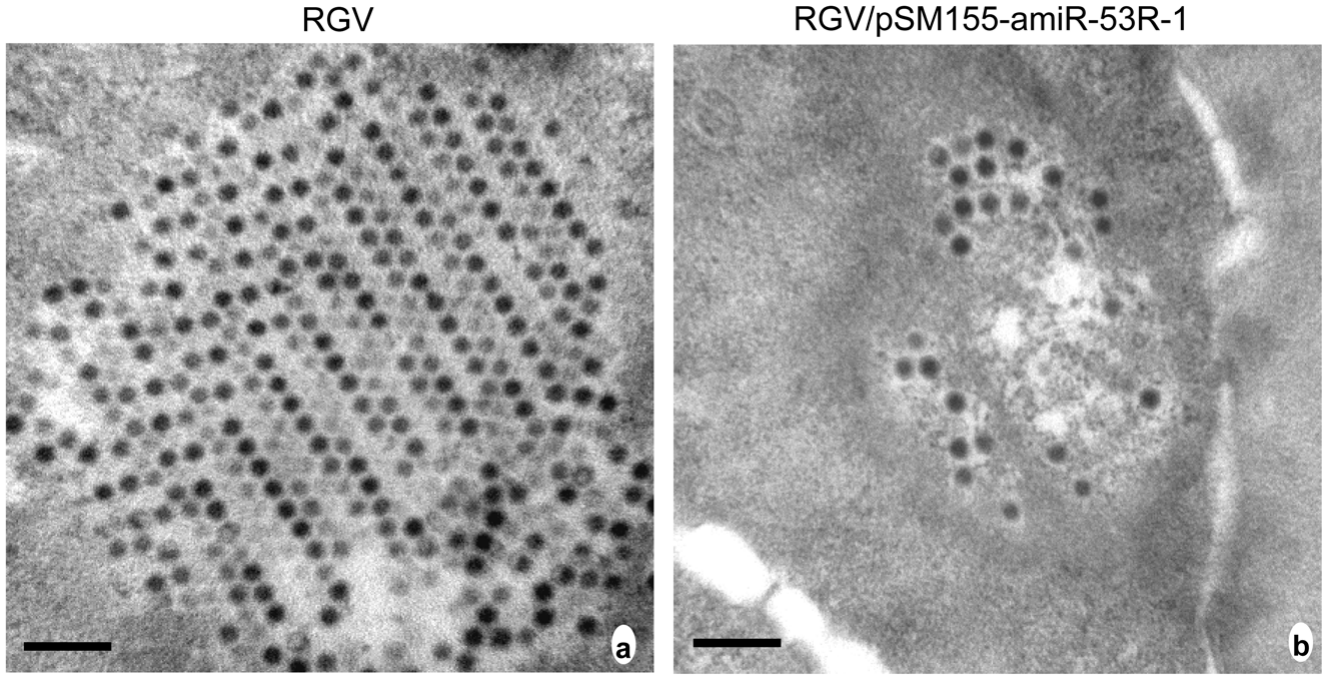
Electron micrograph of RGV. EPC cells were transfected with pSM155-amiR-53R-1 and infected with RGV 24 h post transfection (MOI of 3). After 72 h of RGV infection, cells were collected for electron microscopy. (a) Crystalline aggregation contained large number of regular arranged virions in host cells only infected with RGV. (b) Several sporadic RGV virions with irregular arrangement in host cells transfected with pSM155-amiR-53R-1 and then infected with RGV.

Observation of RGV virions in ultrathin sections of *epithelioma papulosum cyprini* (EPC) cells by electron microscopy showed that larger numbers of RGV virions were assembled and arranged orderly in the cytoplasm of the host cells only infected with RGV (Fig. 4-a). In pSM155-amiR-53R-1 transfected EPC cells, several sporadic RGV virions were present in the cytoplasm with irregular arrangement and the number of virions were significantly decreased (Fig. 4-b).

### RGV resistance by amiR-53R-1

**Figure 5:**
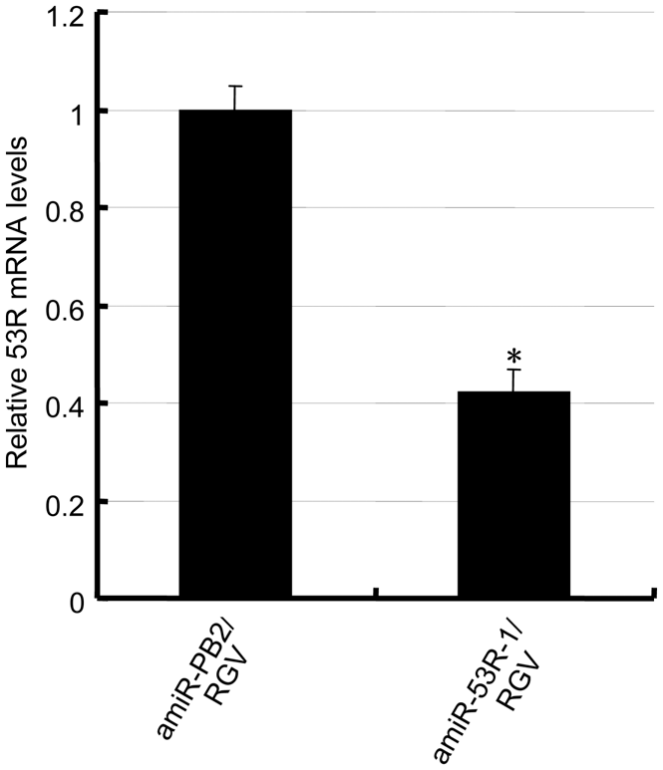
Relative *53R* mRNA levels in RGV infected cells. GCO cells were transfected with pSM155-amiR-53R-1 or pSM155-amiR-PB2, respectively, and infected with RGV 24 h post transfection (MOI of 3). After 72 h of RGV infection, 53R mRNA levels were measured by real-time PCR analysis. The value of pSM155-amiR-53R-1 transfected cells was 0.42±0.05 when the negative control group (transfected with pSM155-amiR-PB2) designated as 1.0. The values represent averages of three independent experiments, with the range indicated (±S.D). *P<0.05 versus control.

It has been shown in above that *53R* silencing and RGV inhibition by pSM155-amiR-53R-1 delayed emergence of CPE, or reduced RGV titer and assembled virions in transfected fish cells. So *53R* expression was assayed as the RGV inhibition efficiency in RGV infected GCO cells that transiently transfected with pSM155-amiR-53R-1 or pSM155-amiR-PB2 by real-time PCR. The data indicated that the *53R* expression was reduced by 58% (FC = 0.42±0.05) at 72 h p.i. of RGV in pSM155-amiR-53R-1 transfected cells with compared to the pSM155-amiR-PB2 transfected cells (Fig. 5).

### No *Mx* response by amiR-53Rs

**Figure 6:**
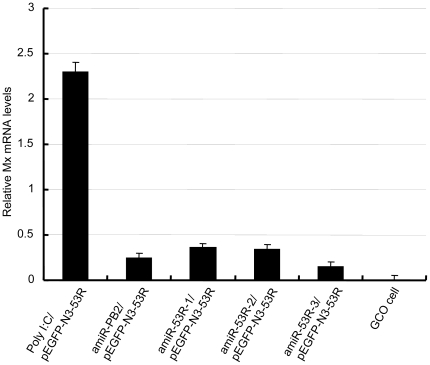
Expression of the *Mx* in different transfected cells. Quantitative real time PCR was used to measure *Mx* mRNA. The value of the control (GCO cell) was designated as 0 (n = 3). Expression of *Mx* gene was not significantly changed when compared to the control except in cells treated with poly I:C. The values represent averages of three independent experiments, with the range indicated (±S.D).

*Mx* is a key component of the interferon response. To determinate whether transfection of GCO cells with plasmids expressing 53R-targeted amiR-53Rs and pEGFP-N3-53R resulted in induction of IFN response, mRNA levels of *Mx* were detected. There were no significant changes in expression of *Mx* gene in GCO cells that were transfected with different pSM155-amiR-53Rs (Fig. 6). These results indicated that the antiviral activity was not induced by *Mx* expression but by RNAi.

## Discussion

Envelope protein gene *53R* has been reported as one of the twenty six core genes existed in all iridoviruses [22]. Present study was the first time that the envelope protein targeted amiRNA was used for silencing an iridovirus efficiently. It revealed that envelope protein was also good target for iridovirus inhibition besides the MCP and offers a new approach for controlling viral infections.

Three amiRNAs targeted to different positions were constructed and used in this study. Two of these, amiR-53R-1 and amiR-53R-3, had the diversity in just one nucleotide in the targeted positions. AmiR-53R-1 targeted sequence was located in positions 36–57 of RGV *53R* whereas amiR-53R-3 targeted 37–58. But the inhibition efficiency of these two amiRNAs was distinctly different. AmiR-53R-1 reduced 53R expression in 74% compared to 35% acquired by amiR-53R-3 when co-transfected with pEGFP-N3-53R. It suggested that amiRNA targeted positions of envelope protein was highly specific. And the selection of specific positions in future amiRNA design was very important.

Single or double stranded RNAs could active the immune response of host cells to resist virus infection [23]. So the amiRNAs may active immune response, especially the interferon (IFN)-related pathways, to inhibit virus. Poly I:C was used in this study as the positive control. It has been reported that poly I:C was a inducer of IFN *in vitro* and *in vivo* [24], [25]. Poly I:C also has been used in fish to induce IFN response [26], [27]. In the poly I:C treated group of the present study, Mx expression was largely up-regulated. So it was considered that Mx gene used in this study was sensitive for IFN. And it was proved in this research that IFN response was not activated apparently by examine the *Mx* expression, which was a key component in IFN response [28]. The pre-amiRNAs used for RGV 53R inhibition had a mismatch at the +1 position and a bulge at the +11, +12 positions in its stem. It's different from the pre-amiRNAs without a mismatch at the +1 position and a bulge at the +12, +13 positions which was found to activate IFN-related pathways [10]. Different *Mx* inducing ability may be due to different structures of the pre-amiRNAs. Moreover, RGV titers obtained in the less active amiRNA treated cells were between those from amiR-53R-1 and negative controls. It indicated that the decrease of RGV titers was mostly attributed to the knock down of *53R* expression.

Inhibition of viral yield was less at 96 hr p.i. than at the two earlier time points. It may be due to the reduction of pSM155-amiR-53R-1 plasmid in transfected cells. The level of plasmids in cells at 96 hr p.i. was less than at the two earlier time points, probably due to the degradation of plasmids with time. So the inhibition effect was less as the time went by.

On the whole, we reported the first iridovirus envelope protein gene targeted amiRNAs that could mediate iridovirus resistance by the specific gene-silence pathway. This system could be used for the inhibition of iridovirus replication and the future development of strategies to control viral diseases in aquaculture. The most highly inhibition efficiency in our study was 58% (obtained by amiR-53R-1). It is slightly lower than the inhibition efficiency by MCP targeted amiRNAs [10]. Therefore, future work should be done to increase the inhibition efficiency of envelope protein gene targeted amiRNAs.

## Materials and methods

### Cell lines and virus

A strain of frog iridovirus, *Rana grylio* virus (RGV), and two fish cell lines, grass carp ovaries (GCO) and *epithelioma papulosum* cyprinid (EPC) cells, were used in these studies (GCO cell and EPC cell were maintained in our laboratory, please see the references) [29], [30]. Cell culture, virus propagation and virus titer determination were performed as described previously [31], [32].

### AmiRNAs device and plasmids construction

Three pairs of oligonucleotides encoding 53R-specific amiRNAs of RGV (referred as amiR-53R-1, amiR-53R-2 and amiR-53R-3) (Table 1), and a pair of oligonucleotides corresponding to PB2 gene of avian influenza virus, AIV (referred as amiR-PB2), were designed using the BLOCK-iT™ RNAi Designer/miR RNAi (http://rnaidesigner.invitrogen.com). Each oligonucleotide pair (“top strand” and “bottom strand” oligos) was annealed and ligated into the pre-miRNA155 (pSM155) vector to create plasmids (pSM155-amiR-53Rs and pSM155-amiR-PB2) capable of producing *53R* and *PB2* encoded pre-amiRNAs in plasmid transfected cells. The pSM155 vector was kindly provided by Prof. De-Yin Guo and Dr. On-Sam Sin. It was based on the backbone of natural miR-155 in which the natural miRNA:miRNA duplex sequence was replaced by the artificial one. Briefly, the natural miR-155 sequence was inserted into the pEGFP-N1 vector under a CMV promoter to create pSM155 vector. The oligonucleotides encoding amiRNA was inserted into the BsmBI site of miR-155 to replace the nature miRNA duplex sequence [19]. The *53R*-expressing plasmid (pEGFP-N3-53R) constructed in previous studies [7] was used in co-transfection experiments to express the target *53R*.

### Transfection

GCO and EPC cells were seeded into 12-well or 6-well cell culture plates using 199 medium containing 5% of FBS 24 h before transfection. Cells were transfected with plasmids using Lipofectamine 2000 (Invitrogen, U.S.A.) following the manufacturer's protocol. The transfection mixtures were removed at 6 h post transfection, and transfected cells were maintained for further processing.

### AmiRNAs expression and anti-RGV activity detection

To select the amiR-53R that has the best inhibition efficiency, the amiR-53Rs were initially tested for sequence-specific silencing on the target 53R gene by employing transient transfection of a plasmid expressing *53R* (pEGFP-N3-53R). GCO cells were co-transfected with pSM155-amiR-53R-1/pEGFP-N3-53R, pSM155-amiR-53R-2/pEGFP-N3-53R and pSM155-amiR- 53R-3/pEGFP-N3-53R, respectively. At 72 h of transfection, total RNA was extracted from transfected cells and reverse transcribed to cDNA for real-time PCR analysis. Cells transfected with pSM155-amiR-PB2/pEGFP-N3-53R were used as negative control.

To elucidate antiviral effect of amiR-53R-1 on RGV replication, the expression of 53R gene was monitored in GCO cells transfected with pSM155-amiR-53R-1 and infected with RGV at an MOI of 3 after 24 h of transfection. The pSM155-amiR-PB2 was used for negative control. At 72 h of p.i, cDNA obtained by method as above were used for real-time PCR analysis.

To assess inhibitory effect of amiR-53R-1 on RGV replication in terms of the production of viral particles, GCO cells transfected with pSM155-amiR-53R-1 and infected with RGV at an MOI of 3 after 24 h of transfection. Cell supernatants were collected at 24 h, 48 h, 72 h and 96 h p.i., respectively. The pSM155-amiR-53R-3 was used as comparison and pSM155-amiR-PB2 as negative control. Monolayer of GCO cells seeded in 96-well plates was inoculated with serial 10-fold dilutions of RGV samples for TCID_50_ detection.

### Electron microscope observation

EPC cells were transfected with pSM155-amiR-53R-1 and infected with RGV at an MOI of 3. Cells were centrifuged at 2000×g 72 h p.i. and the pellets were fixed in 2.5% glutaraldehyde in 0.1 M phosphate buffer (pH = 7.4) for 1 day, rinsed in 0.1 M phosphate buffer containing 1% osmium tetroxide for 1 h at 4°C, dehydrated in a graded ethanol series and embedded in Spurr's resin. The cell pellets were post-fixed with 2% osmium tetroxide in sodium cacodylate buffer for 1 to 2 h, dehydrated in a graded acetone series, and embedded in LR White acrylic resin. Ultra-thin sections were post-stained with 2% uranyl acetate and 3% lead citrate and observed at 100 kV with a JEOL 1230 transmission electron microscope.

### *Mx* expression detection

The expression level of *Mx* was investigated by real time PCR in cells co-transfected with poly (I:C)/pEGFP-N3-53R, pSM155-amiR-PB2/pEGFP-N3-53R, pSM155-amiR-53Rs/pEGFP-N3-53R, respectively. Control cells were transfected only with Lipofectamine 2000 as well as in cells transfected with pEGFP-N3-53R.

### Real-time PCR

RNA extracted by Trizol reagent (Invitrogen, USA) was treated with DNase I and added to the reverse transcription reaction containing 200 U M-MLV reverse transcriptase (promega, USA), 0.5 mM each of dNTP, 1**×**M-MLV reaction buffer, 500 ng of oligo(dT)_15_ Primer and 25 U Rnasin (TOYOBO).

Real-time PCR was performed with 2 µl of cDNA in a final volume of 25 µ1 containing 11.25 µ1 of 2.5**×**RealMasterMix/20**×**SYBR solution(TIANGEN BIOTECH), 300 nM of sense primer and antisense primer. PCR was carried out in the 7300 Fast Real-time PCR System (Applied Biosystems, USA) using the following thermal cycling profile: 95°C for 3 min, followed by 40 cycles of amplification (95°C for 20 s, 58°C for 30 s, 68°C for 45 s). The absorption values of the SYBR Green I in each tube were detected at the end of each cycle. The melting curve analysis of PCR products from 55 to 95°C were also performed after PCR amplification. PCR was performed with the 53R forward primer (5**′**-CATCAGAACGGGAGGACAGA-3**′** **), the 53R reverse primer (5**′**- CGCCGTGTCGTCCTTGTAG-3**′** **), the β-actin forward primer (5′-GATGATGAAATTGCCG CACTG-3′), the β-actin reverse primer (5′-ACCAACCATGACACCCTGATGT-3′), the Mx forward primer (5′-GCTGGAACGGGAGAAGGGAT-3′), and the Mx reverse primer (5′-CGCCGT GTCGTCCTTGTAG-3′). Fragments were 244 bp (*53R*), 120 bp (*β-actin*) and 250 bp (*Mx*), respectively. The Fold Change (FC = 2^−*ΔΔC*T^) in *53R* and *Mx* mRNA expression levels were normalized to those of the β-actin transcripts measured with the same cDNAs. The relative *53R* and *Mx* mRNA expression values in each group were calculated by the mathematical delta–delta method [33] and all samples were run in triplicate.

## References

[1] Williams T (1996). The iridoviruses.. Adv Virus Res.

[2] Zhang QY, Xiao F, Li ZQ, Gui JF, Mao JH (2001). Characterization of an iridovirus from the cultured pig frog *Rana grylio* with lethal syndrome.. Dis Aquat Org.

[3] Zhao Z, Ke F, Gui JF, Zhang QY (2007). Characterization of an early gene encoding for dUTPase in *Rana grylio* virus.. Virus Res.

[4] Zhao Z, Ke F, Shi Y, Zhou GZ, Gui JF (2009). Rana grylio virus thymidine kinase gene: an early geneof iridovirus encoding for a cytoplasmic protein.. Virus Genes.

[5] Sun W, Huang YH, Zhao Z, Gui JF, Zhang QY (2006). Characterization of the *Rana grylio* virus 3beta-hydroxysteroid dehydrogenase and its novel role in suppressing virus-induced cytopathic effect.. Biochem Biophys Res Commun.

[6] Ke F, Zhao Z, Zhang GY (2009). Cloning, expression and subcellular distribution of a *Rana grylio* virus late gene encoding ERV1 homologue.. Mol Biol Rep.

[7] Zhao Z, Ke F, Huang YH, Zhao JG, Gui JF (2008). Identification and characterization of a novel envelope protein in *Rana grylio virus*.. J Gen Virol.

[8] Yeung ML, Bennasser Y, Le SY, Jeang KT (2005). siRNA, miRNA and HIV: promises and challenges.. Cell Res.

[9] Xie JF, Lu L, Deng M, Weng S, Zhu JY (2005). Inhibition of reporter gene and iridovirus-tiger frog virus in fish cell by RNA interference.. Virology.

[10] Dang LT, Kondo H, Aoki T, Hirono I (2008). Engineered virus-encoded pre-microRNA (pre-miRNA) induces sequence-specific antiviral response in addition to nonspecific immunity in a fish cell line: convergence of RNAi-related pathways and IFN-related pathways in antiviral response.. Antiviral Res.

[11] Dang LT, Kondo H, Hirono I, Aoki T (2008). Inhibition of red seabream iridovirus (RSIV) replication by small interferring RNA (siRNA) in a cell culture system.. Antiviral Res.

[12] Denli AM, Tops BB, Plasterk RH, Ketting RF, Hannon GJ (2004). Processing of primary microRNAs by the microprocessor complex.. Nature.

[13] Sui HY, Zhao GY, Huang JD, Jin DY, Yuen KY (2009). Small Interfering RNA Targeting M2 Gene Induces Effective and Long Term Inhibition of Influenza A Virus Replication.. PLoS One.

[14] Du G, Yonekubo J, Zeng Y, Osisami M, Frohman MA (2006). Design of expression vectors for RNA interference based on miRNAs and RNA splicing.. FEBS J.

[15] Stegmeier F, Hu G, Rickles RJ, Hannon GJ, Elledge SJ (2005). A lentiviral microRNA-based system for single-copy polymerase II-regulated RNA interference in mammalian cells.. Proc Natl Acad Sci USA.

[16] Shin KJ, Wall EA, Zavzavadjian JR, Santat LA, Liu J (2006). A single lentiviral vector platform for microRNA-based conditional RNA interference and coordinated transgene expression.. Proc Natl Acad Sci USA.

[17] Zeng Y, Cullen BR (2004). Structural requirements for pre-microRNA binding and nuclear export by Exportin 5.. Nucleic Acids Res.

[18] Boudreau RL, Martins I, Davidson BL (2009). Artificial microRNAs as siRNA shuttles: improved safety as compared to shRNAs *in vitro* and *in vivo*.. Mol Ther.

[19] Onsam Sin (2009). Relationship of gene silencing effects and the structure and targeting sites of intronic spliced artificial miRNAs.. Wunan University Ph.D thesis.

[20] Zamore PD, Tuschl T, Sharp PA, Bartel DP (2000). RNAi: double-stranded RNA directs the ATP-dependent cleavage of mRNA at 21 to 23 nucleotide intervals.. Cell.

[21] Huang XH, Huang YH, Yuan XP, Zhang QY (2006). Electron microscopic examination of the viromatrix of *Rana grylio* virus in a fish cell line.. J Virol Methods.

[22] Eaton HE, Metcalf J, Penny E, Tcherepanov V, Upton C (2007). Comparative genomic analysis of the family Iridoviridae: re-annotating and defining the core set of iridovirus genes.. Virol J.

[23] Dykxhoorn DM, Novina CD, Sharp PA (2003). Killing the messenger: short RNAs that silence gene expression.. Nat Rev Mol Cell Biol.

[24] Magee WE, Griffith MJ (1972). The liver as a site for interferon production in response to poly I:poly C.. Life Sci II.

[25] Manetti R, Annunziato F, Tomasevic L, Gianno V, Parronchi P (1995). Polyinosinic acid: polycytidylic acid promotes T helper type 1-specific immuneresponses by stimulating macrophage production of interferon-alpha and interleukin-12.. Eur J Immunol.

[26] Ooi EL, Hirono I, Aoki T (2006). Functional characterisation of the Japanese flounder, Paralichthys olivaceus, Mx promoter.. Fish Shellfish Immunol.

[27] Jiang J, Zhang YB, Li S, Yu FF, Sun F (2009). Expression regulation and functional characterization of a novel interferon inducible gene Gig2 and its promoter.. Mol Immunol.

[28] Robertsen B (2006). The interferon system of teleost fish.. Fish Shellfish Immunol.

[29] Zhang QY, Ruan HM, Li ZQ, Yuan XP, Gui JF (2003). Infection and propagation of lymphocystis virus isolated from the cultured flounder Paralichthys olivaceus in grass carp cell lines.. Dis Aquat Org.

[30] Fijan N, Sulimanovic D, Berzotti M (1983). Some properties of the *Epithelioma papulosum cuprini* (EPC) cell line from carp (*Cyprinus carpio*).. Ann Virol(Paris).

[31] Zhang QY, Zhao Z, Xiao F, Li ZQ, Gui JF (2006). Molecular characterization of three *Rana grylio* virus (RGV) isolates and *Paralichthys olivaceus* lymphocystis disease virus (LCDV-C) in iridoviruses.. Aquaculture.

[32] Zhang QY, Li ZQ, Gui JF (1999). Studies on morphogenesis and cellular interactions of *Rana grylio* virus in an infected fish cell line.. Aquaculture.

[33] Pfaffl MW (2001). A new mathematical model for relative quantification in real-time RT-PCR.. Nucleic Acids.

